# TMEM184B promotes proliferation, migration and invasion, and inhibits apoptosis in hypopharyngeal squamous cell carcinoma

**DOI:** 10.1111/jcmm.17572

**Published:** 2022-10-18

**Authors:** Yun Lin, Dayu Liu, Xuexin Li, Yan Ma, Xinliang Pan

**Affiliations:** ^1^ Department of Otolaryngology Qilu Hospital of Shandong University Qingdao China; ^2^ NHC Key Laboratory of Otorhinolaryngology (Shandong University) Jinan China

**Keywords:** apoptosis inhibition, hypopharyngeal squamous cell carcinoma, invasion, metastasis, migration, proliferation, TMEM184B, transmembrane protein

## Abstract

Several members of the transmembrane protein family are associated with the biological processes of human malignancies; however, the expression pattern and biological function of one family member, TMEM184B, in hypopharyngeal squamous cell carcinoma (HPSCC) are not fully understood. The expression between HPSCC tumours and adjacent normal tissues was determined by the Immunohistochemistry (IHC). A bioinformatics analysis was performed to verify the expression pattern of TMEM184B in The Cancer Genome Atlas (TCGA) and Gene Expression Omnibus (GEO) databases. Furthermore, in vitro assays on cell proliferation, invasion, migration and in vivo experiments on tumour growth and apoptosis of TMEM184B in HPSCC were performed. We found that the HPSCC tissues had a significantly higher expression of TMEM184B than the adjacent normal tissues. Bioinformatics analysis confirmed the different expression of TMEM184B expression in HPSCC. Furthermore, in vitro and in vivo experiments demonstrated that TMEM184B promotes HPSCC cell growth, cell invasion and migration in FaDu cells, whereas flow cytometry assay showed that TMEM184B inhibited cell apoptosis. Our study revealed for the first time that TMEM184B might serve an oncogenic function in HPSCC and could be a potential diagnostic biomarker and therapeutic target for HPSCC.

## 1 INTRODUCTION

Hypopharyngeal squamous cell carcinoma (HPSCC) accounts for approximately 3% of all head and neck squamous cell carcinoma (HNSCC). The incidence of HPSCC has increased during the past three decades.^1^, ^2^ Although HPSCC is relatively uncommon, it is one of aggressive and lethal forms among HNSCCs. HPSCC is characterized by its wide submucosal spread, early lymph node metastasis, distal metastasis and diagnosis with a later stage. Although improvements have been made to treatments, such as surgery, radiotherapy, chemotherapy, immunotherapy and in their combination in recent decades,^3^, ^4^ the 5 years overall survival of HPSCC remains approximately 30%–35%.^5^ As hypopharynx is anatomically adjacent to the larynx, the advanced‐stage HPSCC may severely impair the structure and function of the larynx, leading to the substantial damage to patients' breath, voice and swallowing functions, thereby adversely affecting their quality of life. Therefore, the improvement of survival and preservation of organs remains important for treatment of HPSCC^6^. Since the low prognosis of HPSCC is largely attributed to the lack of early diagnosis, the development of new diagnostic and prognostic biomarkers^7^ may help improve survival and quality of life for these patients.

TMEM184B, also known as NDC1, belongs to the transmembrane protein (TMEM) family. TMEMs are a group of proteins located in the phospholipid bilayer membrane of cells and organelles,^8^ and they may play different roles in various tissues and subcellular tissues. For example, TMEM48 is an important component of the nuclear pore complex and plays an important role in maintaining the integrity of nuclear pore and normal function of nuclear‐cytoplasmic transport.^9^ TMEM97, also known as MAC30, is an insulin‐like growth factor‐binding protein in the cytoplasm. This protein is expressed in the foetal liver and participates in the development and differentiation of liver.^10^ TMEMs are abnormally expressed in many malignancies.^11^ Their altered expression is significantly correlated with tumour prognosis, metastasis and drug resistance.^12^, ^13^ Moreover, these proteins are involved in different physiological stages of malignancies, such as tumorigenesis,^14^ growth, adhesion, invasion, apoptosis^15^ and metastasis.^8^ Genetic and epigenetic changes of these genes^16^ can affect their expression levels. TMEM158 is overexpressed in ovarian cancer and may promote the occurrence, proliferation, adhesion and invasion in ovarian cancer.^14^ TMEM97 (MAC30) is highly expressed in breast and hepatocellular carcinomas. It inhibits apoptosis and epithelial‐mesenchymal transition (EMT) in breast cancer and promotes the formation, proliferation and invasion of liver cancer cells.^17^ Knockdown of TMEM14A in ovarian cancer cell lines can affect activation of TGF‐β pathway and inhibit the proliferation of ovarian cancer cells.^15^

In our previous study, we found that TMEM184B was significantly overexpressed in HPSCC tissues than that in the adjacent normal mucosa by sequencing. Furthermore, we searched the public database (The Cancer Genome Atlas) and literatures and found that TMEM184B is one of novel TMEM proteins whose role and function have not been fully elucidated in human cancers, including HPSCC. In this study, we have performed in vitro and in vivo experiments for the first time to investigate the roles of TMEM184B in HPSCC.

## 2 MATERIALS AND METHODS

### 2.1 Patients and specimens

A total of 40 formalin‐fixed, paraffin‐embedded (FFPE) HPSCC specimens and the paired normal tissues (tumour‐adjacent) were obtained from tumour bank in the Pathology Department of the Qilu Hospital(Qingdao) of Shandong University between 2015 and 2016. The specimens were collected and used according to the ethical guidelines and procedures approved by the Research Ethics Review Board of the Qilu Hospital (Qingdao) at the Shandong University. The staging of HPSCC was defined in accordance with American Joint Committee on Cancer TNM staging system. The characteristics of 40 patients were listed in Table 1 **.**

**TABLE 1:** Demographic and clinicopathological characteristics of patients with HPSCC (*N* = 40)

Characteristics	Numbers of patients/number analysis	%
Age (median, range)	60(45–87) [years]	
Gender		
Female	2	5.0
Male	38	95.0
Smoking		
Never	3	7.5
Ever	37	92.5
Drinking		
Never	6	15.0
Ever	34	85.0
T category		
T1‐T2	12	30.0
T3‐T4	28	70.0
N category		
N0‐N1	25	62.5
N2‐N3	15	37.5
Clinical Stage		
I‐II	4	10.0
III‐IV	36	90.0
Differentiation Grade		
Well	3	7.5
Moderate	11	27.5
Poor	26	65.0

### 2.2 Immunohistochemistry (IHC)

The tissue sections were initially deparaffinized, hydrated, heated in EDTA (pH 8.0) and then incubated with 3% hydrogen peroxide for 10 min for antigen retrieval. The reaction of TMEM184B rabbit monoclonal antibody (1:500; PA‐5‐20,932 [Thermofish]) was evaluated for 1 h at room temperature, followed by incubation with goat anti‐rabbit biotin‐conjugated IgG (1:500; PA‐5‐20,932 [Thermofish]). The slides were stained with DAB (Shanghai Long Island Biotec. Co., LTD) and haematoxylin (BASO). The IHC signals were obtained from positively stained cells.

### 2.3 Bioinformatics analysis

For verification, we performed an analysis on the expression profile of TMEM184B of HPSCC with the data from online publicly accessible databases, including The Cancer Genome Atlas (TCGA) and Gene Expression Omnibus (GEO) under the accession number GSE58911. The primary data were Log2 standardized for subsequent statistical analysis.

### 2.4 Cell culture and transfection

The FaDu cell line was purchased from the Cell Bank of the Chinese Academy of Sciences. The cells were cultured in Dulbecco's modified Eagle's medium (DMEM; Invitrogen) supplemented with 10% foetal bovine serum (FBS; Gibco), 1% penicillin and streptomycin mixtures in a humidified cell incubator at 37°C with 5% CO_2_.

The RNA interference sequence targeting TMEM184B was designed and cloned into the GV115 lentiviral vector system (GeneChem Co.,). The scrambled sequence (TTCTCCGAACGTGTCACGT) was used as the negative control. The vectors were then cotransfected into the competent E. coli cells. The constructs were subsequently cotransfected into HEK‐293 T cells with lentiviral packaging vectors using Lipofectamine 2000 (Invitrogen Life Technologies) according to the manufacturer's instructions. The viruses were collected at 48 h after transfection and used to infect FaDu cells. All assays were performed at 48 h after infection. The infection efficiency was evaluated using the real‐time PCR and Western blotting.

### 2.5 Reverse transcription and Real‐time PCR

Total RNA was isolated from FaDu cells using TRI Reagent (SuperfecTRI). A 2 μg of the total RNA was reversely transcribed to synthesize cDNA using the M‐MLV Reagent Kit (Promega). The qRT‐PCR was performed on a LightCycler 480 II real‐time PCR system (Roche) using SYBR Premix EX Taq (TAKARA). The primers for TMEM184B were as follows: forward, 5'‐GGAAAGACTTATTCCATCGG‐3'; reverse,5'‐AGAAGAGGAAGAGGGCGTAG‐3'. The primers for the internal control gene GAPDH were as follows: forward, 5'‐TGACTTCAACAGCGACACCCA‐3'; reverse, 5'‐CACCCTGTTGCTGTAGCCAAA‐3'.The mRNA expression levels were calculated using the comparative ΔΔCt method, and fold changes were analysed using 2^−ΔΔCt^.

### 2.6 Western blotting

Total proteins were extracted from FaDu cells with or without infection, resolved on 12% SDS‐PAGE and transferred to PVDF membranes. Rabbit monoclonal antibodies for TMEM184B (Santa, 1:100) and GAPDH (Abcam, 1:10000) were used in Western blotting. The samples were then incubated with Horseradish Peroxidase‐conjugated goat anti‐rabbit secondary antibodies (Aspen, 1:10000). The protein bands were visualized by an electrochemiluminescence kit (CST). The bands in Western blotting were quantified from three replicates.

### 2.7 Cell growth assay by the Celigo system

The transfection efficiency of knockdown of the target gene was determined using RT‐qPCR and Western blotting. The three groups were established: target cells transfected with the negative control virus (shCtrl), target cells transfected with TMEM184B shRNA1 virus (shTMEM184B‐1) and target cells transfected with TMEM184B the shRNA2 virus (shTMEM184B‐2). Then, the cells were cultured at 37°C in a 5% CO_2_ incubator at a volume of 100 μl/well. From the second day after plating, the cell counts were detected using a Celigo Image Cytometer (Celigo, Nexcelom) once a day for 5 days, and the number of cells with green fluorescence was accurately calculated by adjusting the input parameters of the Celigo analysis settings. Data analysis and the plot of the 5 days cell growth curve were performed.

### 2.8 Cell cycle and apoptosis assays by the flow cytometry

The cell apoptosis and cell cycle detection were measured using the flow cytometry (Becton‐Dickinson). The three groups were established as previously mentioned (shCtrl, shTMEM184B‐1 and shTMEM184B‐2, respectively). The cells were seeded onto the 6‐well plates. The cells were stained with 10 μl annexin V‐APC fluorescein isothiocyanate (annexin V‐FITC) for 10 min in the dark for apoptosis detection, as well as with 800 μl of propidium iodide (PI, 5 μg/ml) under the same parameters. The fluorescence signals were evaluated using the flow cytometry.

### 2.9 Cell proliferation assay

The cell proliferation was measured using a Cell Counting Kit‐8 (CCK‐8, Dojindo) according to the manufacturer's instructions. The three groups were established as previously described (shCtrl, shTMEM184B‐1 and shTMEM184B‐2, respectively). The cells were seeded in a 96‐well plate and cultivated at 37°C in a 5% CO_2_ incubator. A total of 100 ml of CCK‐8 solution were added on the second day after the plate spread. The absorbance was measured at 450 nm after 4 h using a microplate reader (Tecan Infinite).

### 2.10 Cell migration assay

An OrisTM obstruction (Platypus Technologies) was sterilized and placed in the 96‐well plates. The transfected cells were set as previously described (shCtrl, shTMEM184B‐1 and shTMEM184B‐2, respectively). For each group, a total of 3–5 × 10^6^ cells were seeded in the 96‐well plates and grown overnight until they reached 90% coverage or above. The OrisTM obstruction (Platypus Technologies) was removed on the second day, and the cells were cultivated at 37°C in a 5% CO_2_ incubator. The plates were scanned at 8,16 and 24 h, respectively, to accurately calculate the area of cells with green fluorescence by adjusting the input parameters of the Celigo analysis. The migration rate was defined as the percentage of cell coverage ratio to “0 hour” fixed area cell coverage ratio at different time points. The data were collected for further calculation and analysis.

### 2.11 Cell invasion assay

Cell invasion assay was performed using the Transwell cell culture chambers coated with Matrigel (Corning), according to the manufacturer's instructions. Following group setting (shCtrl, shTMEM184B‐1 and shTMEM184B‐2, respectively), a total of 1.0 × 10^5^ of transfected cells were seeded in the upper chamber with a serum‐free medium. A 600 μl DMEM containing 10% FBS was added to the lower chamber as a chemoattractant. The cells were incubated at 37°C in an incubator with 5% CO_2_ for approximately 48 h (the time might be adjusted according to the specific situation if needed). The migratory cells were fixed with 4% paraformaldehyde for 30 min and stained with 0.5% crystal violet solution (Shanghai Yuan ye Bio‐Technology Co.) for 3 min. The cells remaining in the upper chambers were removed using the cotton swab. The migratory cells were imaged at 100× and 200× magnification in five random fields per well and counted under a light microscope (Olympus Corp.).

### 2.12 Animal experiments

The FaDu cells were transfected with TMEM184B‐RNAi lentivirus and the negative control (NC). A total of 4 × 10^6^ FaDu cells in the logarithmic phase were subcutaneously injected into the right armpit of 20 four‐week‐old female nude mice (10 mice per group). From the 15th day after injection, the weight of the mice and the length of the tumours were measured twice a week. On the 28th day after injection, the mice were euthanized and the tumours were weighed. All experimental procedures involving animals complied with the ARRIVE guidelines and were performed in accordance with the EU Directive 2010/63/EU for animal experiments. These procedures were approved by the Qilu Hospital of Shandong University (Qingdao) Animal Care Commission.

### 2.13 Statistical analysis

All experiments in this study were repeated at least three times. Data are presented as mean ± SD and were analysed using the SPSS software (version 26.0). Continuous variables were tested using paired or unpaired Student's t‐tests, whereas the chi‐square tests were used for categorical comparisons. Statistical significance was set at *p* values <0.05.

## 3 RESULTS

### 3.1 TMEM184B expression in HPSCC

**FIGURE 1:**
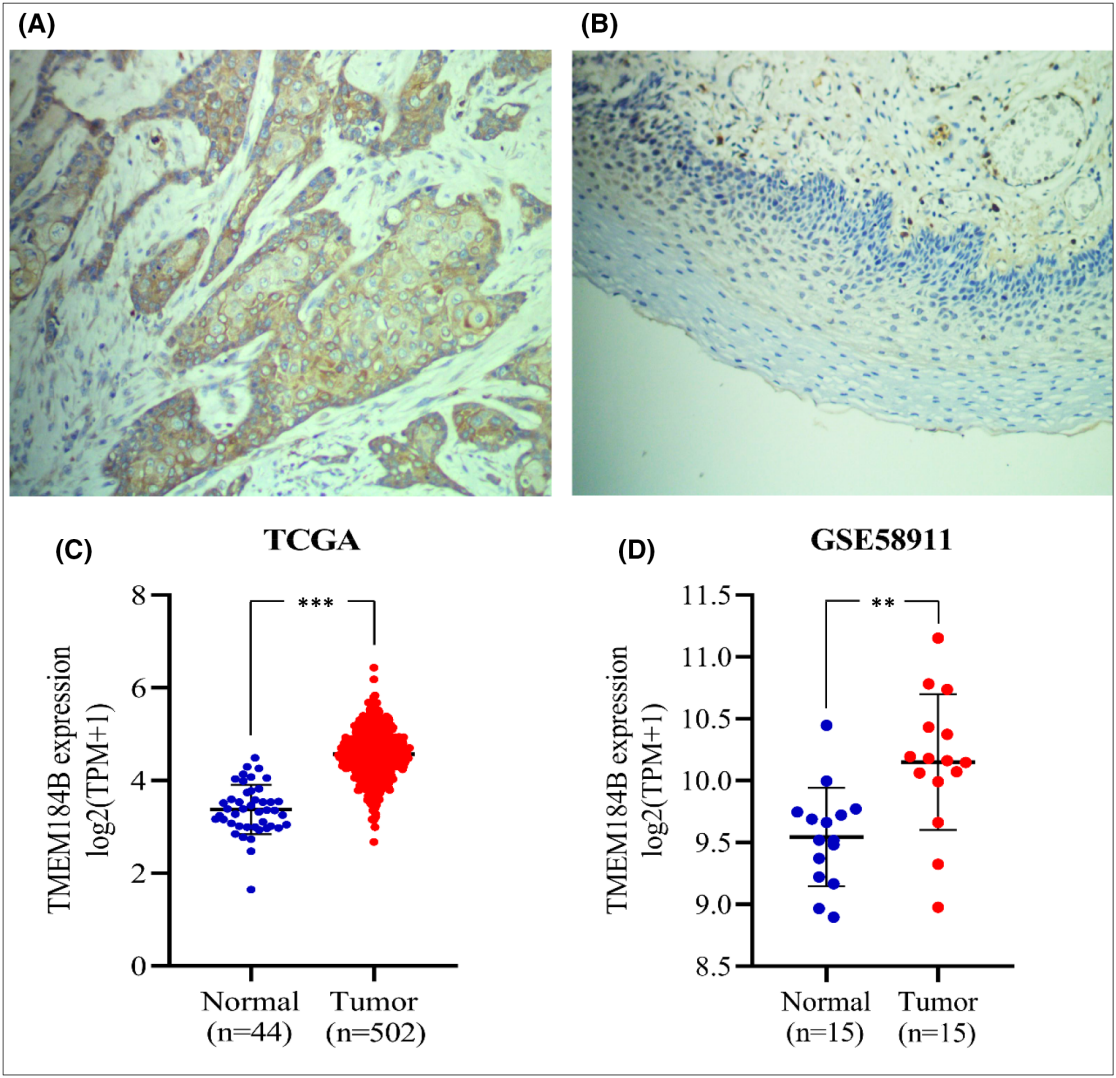
TMEM184B expression in HPSCC tissues. The expression level of TMEM184B in HPSCC tissues (A) compared with that in normal tissues (B) by immunohistochemistry. All images are at 200X magnification. The mRNA expression levels of TMEM184B are significantly higher in HPSCC tissues compared with those in normal tissues from TCGA (C) and GES58911 (D) databases. **p < 0.01, ***p < 0.001

The IHC denotes the expression level of TMEM184B in 40 cancerous tissues **(**Figure 1A **)** and paired normal adjacent mucosa **(**Figure 1B **)** of HPSCC patients; the expression of TMEM184B is significantly higher in cancerous tissues than in normal tissues. Furthermore, a bioinformatics analysis was performed to verify the expression pattern of TMEM184B between cancerous and normal tissues in HPSCC from TCGA and GSE58911 public datasets. Compared with normal tissues, a similar significant change in TMEM184B expression in cancerous tissues was observed in HPSCC tumours in both the TCGA (Figure 1C) and GSE58911 databases (Figure 1D).

### 3.2 Inhibition of TMEM184B knockdown in cell growth of HPSCC in vitro and in vivo

**FIGURE 2:**
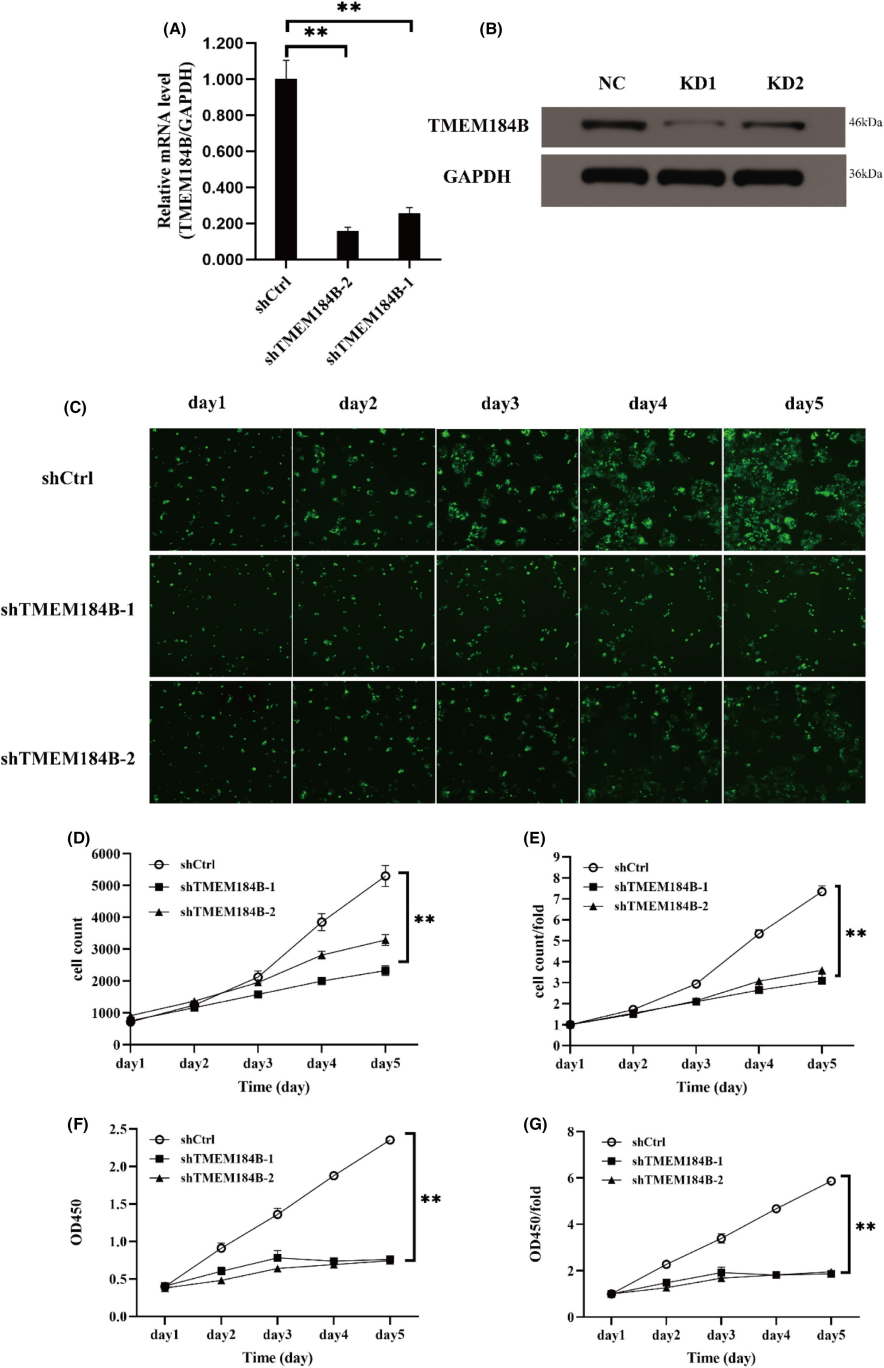
Downregulation of TMEM184B in inhibition of cell growth in FaDu cells. The qRT‐PCR (A) and Western blot analysis (B) to evaluate the infection efficiency. (C) The images presented the growth of Fadu cells for 5 days after transfection by Celigo cell counting assay. (D) The trend of cell count. (E) The trend of fold changes of cells. (F) The absolute value and (G) the fold changes of Fadu cell proliferation detected by CCK‐8 assay every 24 h for 5 days after transfection. *p < 0.05, **p < 0.01 (NC: control; KD1: knockdown of TMEM184B‐1; KD2:knockdown of TMEM184B‐2)

A series of in vitro and in vivo experiments were performed to investigate the correlation between cell growth and proliferation and TMEM184B expression in HPSCC. The expression of TMEM184B was downregulated through mRNA interference, and its efficiency was verified using the RT‐PCR (Figure 2A) and Western blotting (Figure 2B).

After transfection with the shRNA lentivirus, the TMEM184B mRNA expression in FaDu cells was significantly downregulated compared to that in the control group. This downregulation of mRNA led to 50.4% and 42.4% decreases in cell counts (Figure 2D **)** (*p* < 0.05) in the shTMEM184B‐1 and shTMEM184B‐2 groups, respectively, at 4 days after transfection using the Celigo system (Figure 2C). On the 5th day, 58.0% and 51.2% decrease in cell fold change (Figure 2E) (*p* < 0.05) was observed in the two respective experimental groups compared to those in the control group.

In the CCK‐8 assay, the OD value (Figure 2F) and fold change (Figure 2G) of FaDu cells in the shTMEM184B‐1 and shTMEM184B‐2 groups were significantly lower than those in the control group after 4 days of shRNA lentivirus infection. These results indicate that the downregulation of TMEM184B significantly inhibited the proliferation of HPSCC in vitro.

**FIGURE 3:**
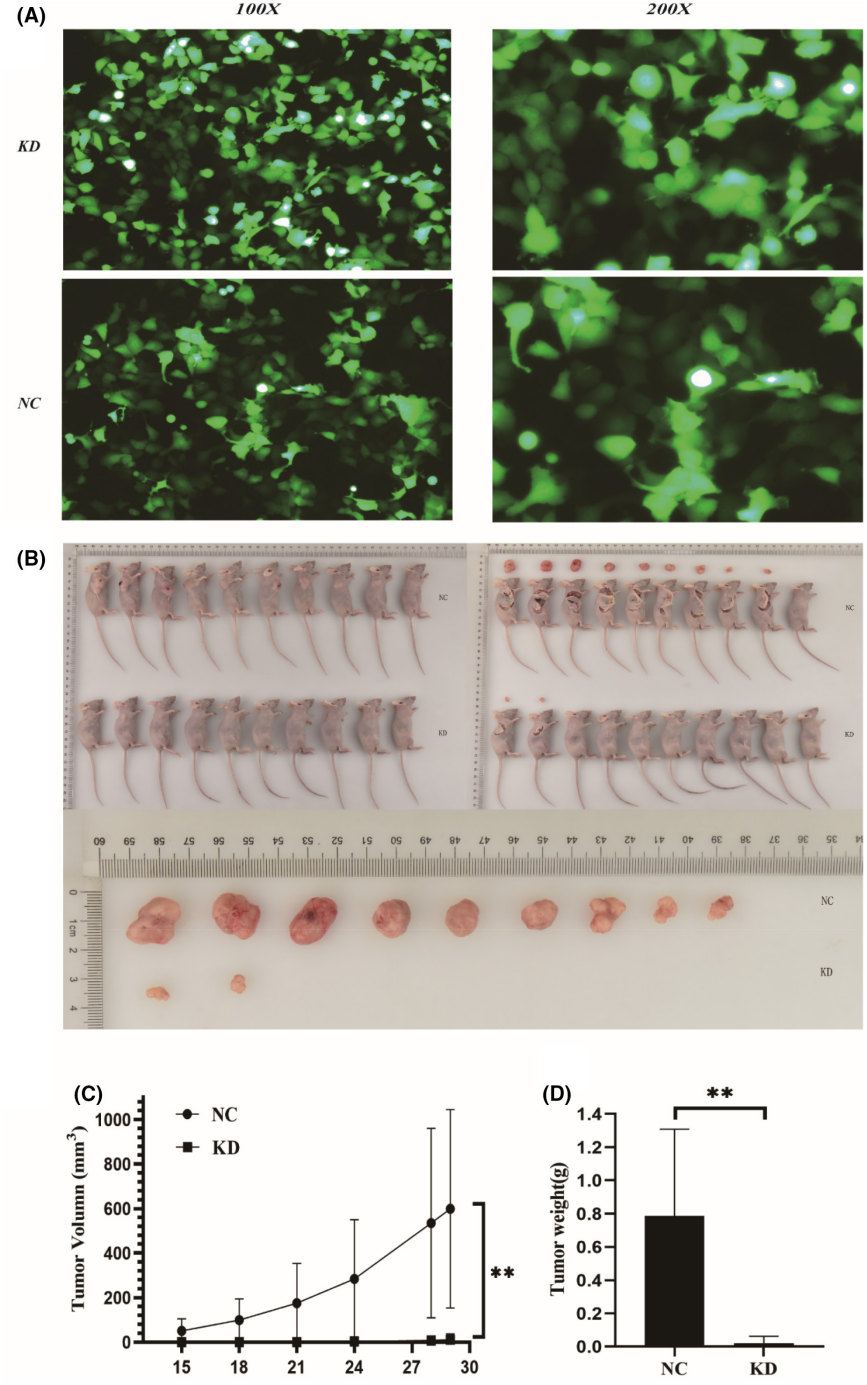
Downregulation of TMEM184B inhibiting tumour growth in vivo. (A) Representative images of transfected FaDu cells by lentivirus beforeinoculation. (B) Representative images of xenograft tumours in nude mice. (C) Averaged volume of the tumour assessed after 15 days of injection and completed at 32 days. (D) Averaged weight of tumours assessed at 32 days after injection. *p < 0.05, **p < 0.01 (NC: control; KD: knockdown of TMEM184B)

To investigate the effect of TMEM184B on HPSCC tumour growth in vivo, the FaDu cells, which were transfected and stably expressed sh‐TMEM184B or NC (Figure 3A), were injected into nude mice (Figure 3B). From the 15th day after injection, the weight of the mice and the volume of the tumours were measured twice a week. In this experiment, a significant reduction in averaged values of tumour volume (Figure 3C) and tumour weight (Figure 3D) was observed in the shTMEM184B group compared to that in the control group. These results suggest that downregulation of TMEM184B inhibits the growth of HPSCC in vivo.

### 3.3 Promotion of TMEM184B knockdown in apoptosis of HPSCC in vitro

**FIGURE 4:**
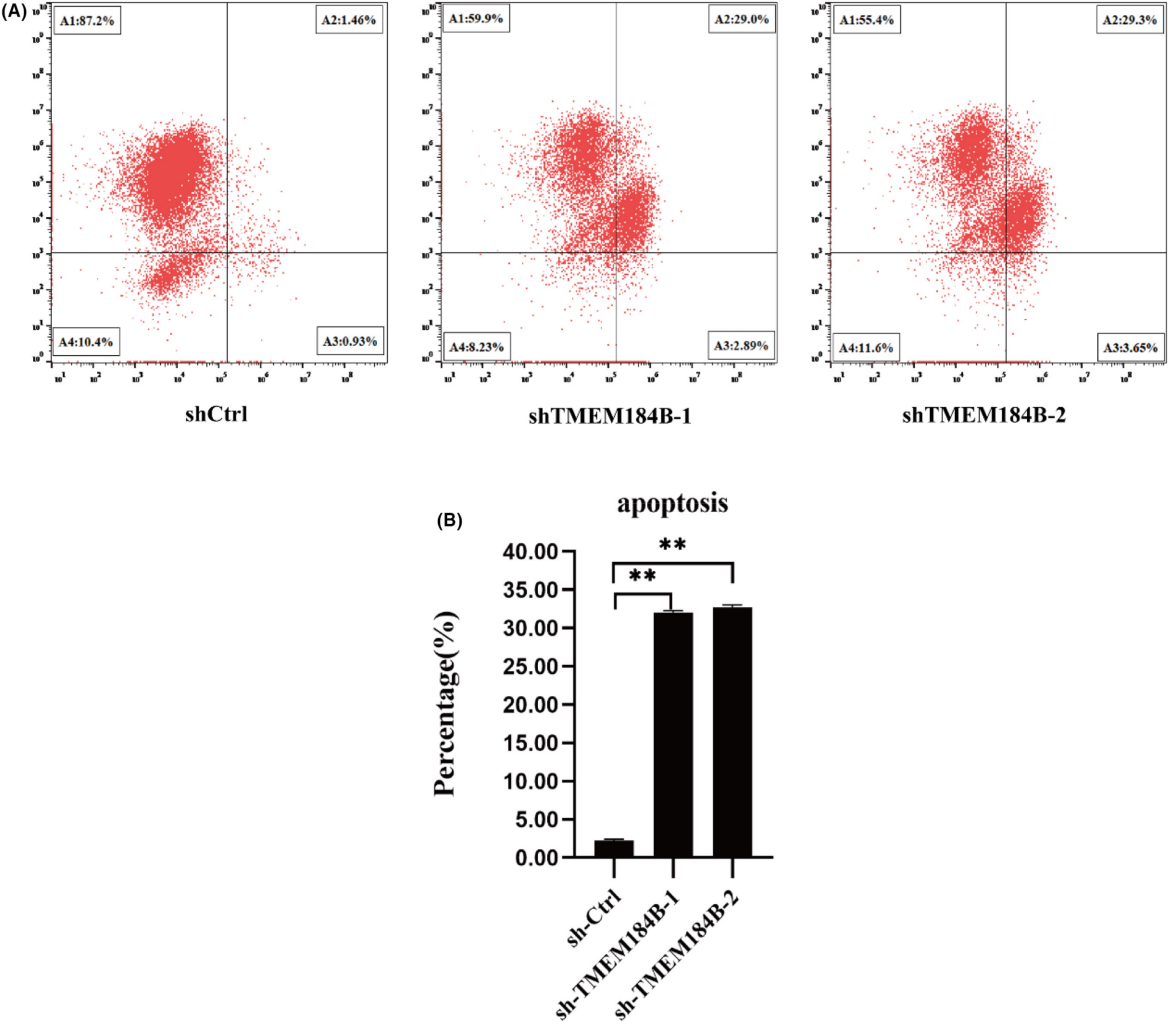
Downregulation of TMEM184B‐induced apoptosis of FaDu cells. (A) FaDu cells staining positive for Annexin‐V APS having undergone apoptosis. (B) Representative histograms depicting apoptosis of FaDu cells. (NC: control; KD1: knockdown of TMEM184B‐1; KD2 knockdown of TMEM184B‐2). **p < 0.01

Flow cytometry was performed to investigate TMEM184B‐mediated cell apoptosis. As shown in Figure 4A and Figure 4B, the apoptotic rate of TMEM184B‐downregulated cells increased from 2.21% to 32.06% and 32.71% in the shTMEM184B‐1 and shTMEM184B‐2 groups, respectively. These results demonstrate that TMEM184B inhibits the FaDu cell apoptosis in vitro, which is consistent with our finding that TMEM184B promotes the growth of FaDu cells.

### 3.4 Inhibition of downregulation of TMEM184B in cell invasion and migration of HPSCC

**FIGURE 5:**
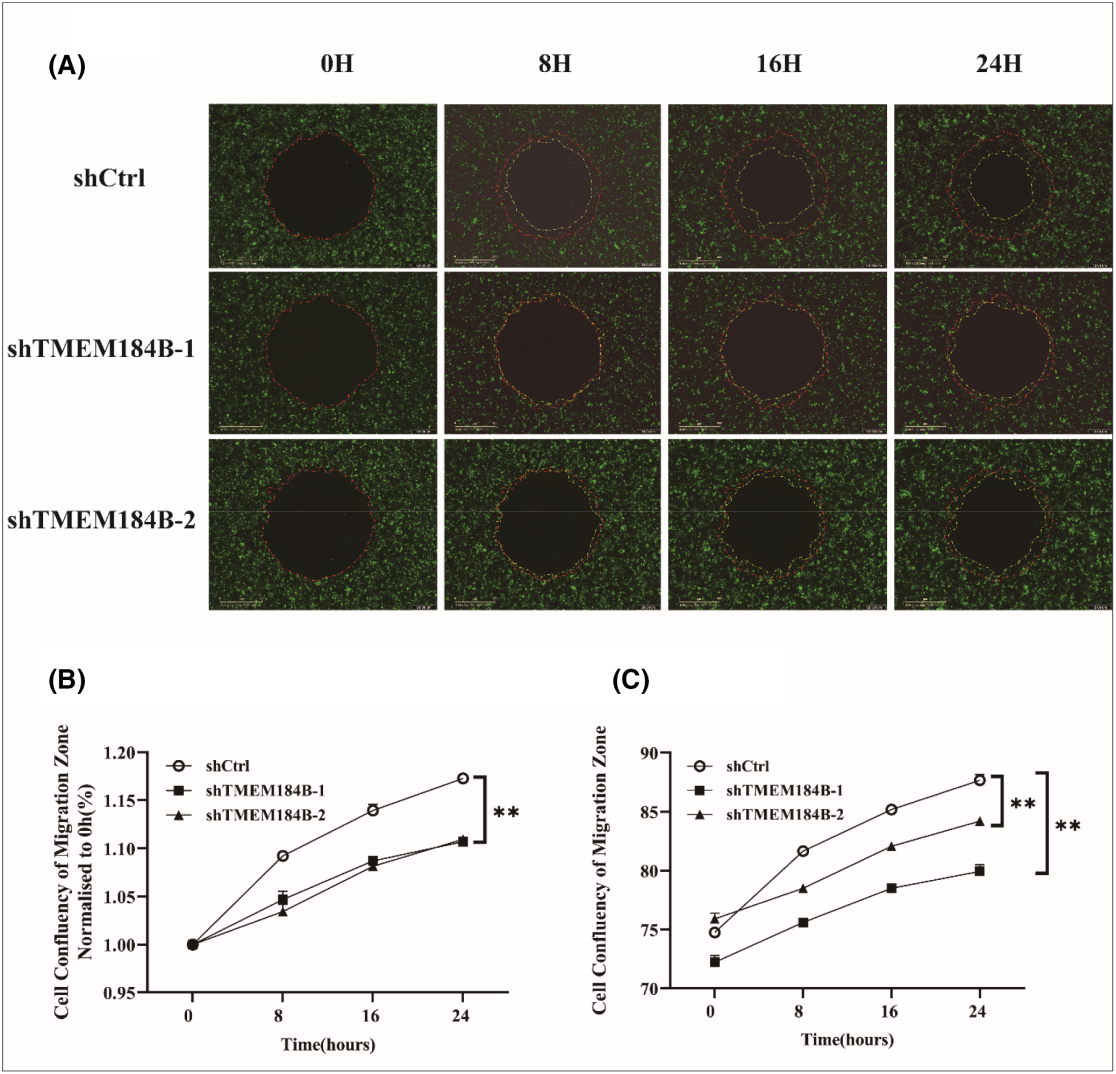
Downregulation of TMEM184B significantly inhibits the migration ability of FaDu cells. A wound‐healing assay using an Oris™ plate was performed to assess the migration ability of FaDu cells after transfection with TMEM184B‐siRNA (i.e. shTMEM184B‐1 and shTMEM184B‐2) and that of FaDu cells transfected with a scrambled control. The boundary of the area covered by the cells at 0 h was outlined by the red dashed lines in each group, and the new boundaries were indicated by the yellow dashed lines at 8, 16 and 24 h, respectively (A). Line charts showed the cell confluency of migration zone normalized to 0 h (B) and the percentage of cell confluency at different time points (C) in each group. **p < 0.01.

**FIGURE 6:**
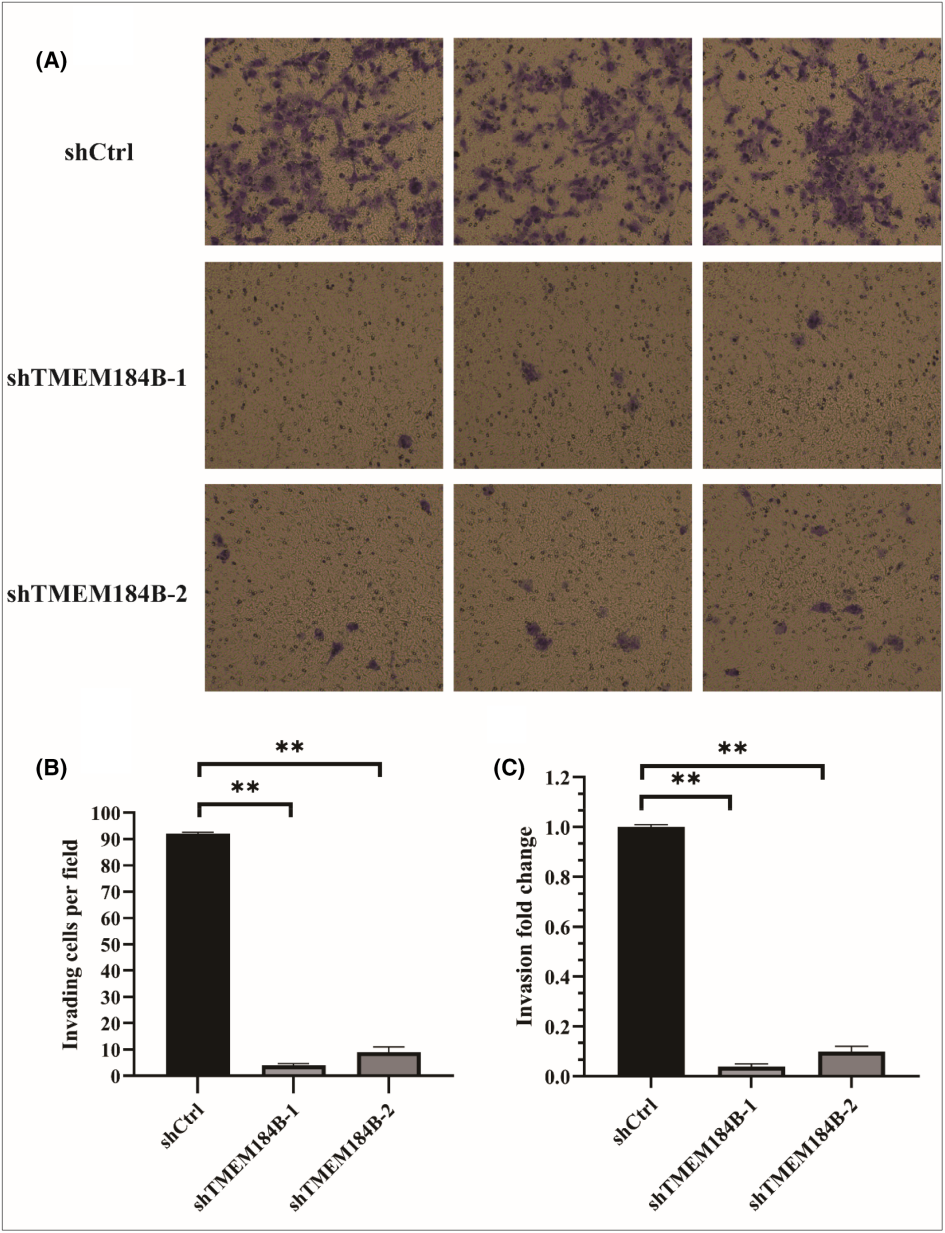
Downregulation of TMEM184B inhibits invasion ability of FaDu cells. A transwell assay was conducted to evaluate the invasive ability of FaDu cells transfected with TMEM184B‐siRNA (i.e. shTMEM184B‐1 and shTMEM184B‐2) and that of FaDu cells transfected with scrambled control. The number of invading cells in the control group was significantly higher than that in shTMEM184B‐1 and shTMEM184B‐2 groups (A). Both the number of invading cells per field (B) and fold changes of invading cells (C) in the shTMEM184B‐1 and shTMEM184B‐2 groups were significantly lower than those in the control group.**p < 0.01

Cell migration was performed using an OrisTM plate (Platypus Technologies). The boundary of the area covered by the cells at 0 h was outlined by the red dashed lines in each group, and the new boundaries were indicated by the yellow dashed lines at 8,16 and 24 h, respectively (Figure 5A). The cell confluency of migration zone normalized to 0 h in each group was shown in Figure 5B **,** and the percentage of cell confluence at different time points in each group was shown in Figure 5C. Compared to the control group, the cell coverage rates in the shTMEM184B‐1 and shTMEM184B‐2 groups were significantly lower at 8,16 and 24 h, respectively, after incubation *(p* < 0.05) (Figure 5). Migration was significantly inhibited in the shTMEM184B‐1 and shTMEM184B‐2 groups (*p* < 0.05). For the Transwell invasion assay, the number of invading cells in the control group was significantly higher than those in shTMEM184B‐1 and shTMEM184B‐2 groups **(**Figure 6A **)**. Both the number of invading cells per field **(**Figure 6B **)** and fold changes of invading cells **(**Figure 6C **)** in the shTMEM184B‐1 and shTMEM184B‐2 groups were significantly lower than those in the control group. These results implied that cell migration and invasive ability were inhibited following the downregulation of TMEM184B in HPSCC.

## 4 DISCUSSION

In this study, we found that TMEM184B may play an oncogenic role in HPSCC. Our results revealed that TMEM184B was highly expressed in HPSCC tissues compared with adjacent normal tissues in both our patients and TCGA database. We investigated the effect of TMEM184B on the phenotype of FaDu cells in vitro*,* indicating that TMEM184B inhibited apoptosis of FaDu cells and promoted their growth, proliferation, migration and invasion. Moreover, our in vivo experiments demonstrated that TMEM184B promoted the proliferation of HPSCC cells. Taken together, these results from the current study provide evidence that TMEM184B plays an oncogenic role in the growth, proliferation, invasion and metastasis of HPSCC, while the biological mechanisms are yet to be explored.

The TMEM family members are functionally diverse and distributed in a variety of cell membranes.^18^, ^19^ Previous studies have confirmed that several proteins in the TMEM family are abnormally expressed in malignant tumours. Altered TMEM protein expression is usually associated with tumour prognosis, metastasis and drug resistance. They may also affect other biological processes, such as tumorigenesis, proliferation, apoptosis, invasion, metastasis, through intercellular adhesion molecules (ICAM),^14^ transforming growth factors(TGF), Wnt/β‐catenin,^20^ PI3K‐Akt, and other pathways via methylation modification and immune regulation.^21^

TMEM184B, also known as FM08, HS5O6A, C22orf5 and HSPC25, is located on chromosome22q13.1. It contains 13 exosomes and is ubiquitous in the placenta, fat and other 25 tissues. The functional study of TMEM184B remains in its early stages, while previous studies have suggested that TMEM184B may play multiple roles in different diseases in different tissues. Bhattacharya et al.^22^ found that TMEM184B is highly expressed in the nervous system with axon degeneration and neuromuscular junction maintenance in mice, suggesting that TMEM184B strongly influences the regulation of axonal degeneration and peripheral nervous system morphology and function. Larsen et al^23^ found that TMEM184B promoted adult somatosensation through developmental Wnt signalling pathway. Rasmussen et al^24^ noted that TMEM184B might regulate ibuprofen uptake by type I cells of Madin‐Darby canine kidneys (MDCK) under hyperosmotic conditions and was regulated by the transcription factor Nfat5. A bioinformatics analysis by Xia et al^25^ revealed that TMEM184B is one of the five hub genes involved in the progression of ANCA‐GN through immune‐related signalling pathways. Similarly, Ji et al^26^ found that TMEM184B might crucially influence the occurrence and development of coronary artery disease by participating in the OIP5‐AS1‐miR‐25‐3p‐TMEM184B ceRNA regulatory network. The roles of TMEM184B in the biological processes of cancer are also gaining attention. In a study on the formation mechanism of piWIL2‐induced cancer stem cells (Piwil‐iCSCs) by Tan et al.,^27^ TMEM184B formed a fusion gene, tMEM184B‐DMC1, with DMC1, and might be related to the occurrence of cancer stem cells. Sara et al.^28^ found that the single nucleotide polymorphisms (SNP) rs7289126 in TMEM184B were associated with both per cent density (PD) and dense area (DA) in mammograms and the risk of breast cancer. In a study of miRNA‐26a/b in oral SCC, Fukumoto et al.^29^ reported that the silencing of TMEM184B could inhibit the migration and invasive ability of cancer cells and regulate gene expression in the actin cytoskeleton pathway.

In summary, the findings from the current study suggest that TMEM184B may represent a new biomarker and therapeutic target for HPSCC, while these findings should be verified in other HPSCC cell lines. However, currently, the FaDu cell line is the only one available for HPSCC in China, and we will validate our results once other HPSCC cell lines become available in our future studies. Furthermore, our future investigation is needed to focus on effect of TMEM184B on outcome and the mechanisms underlying these functions of TMEM184B in HPSCC.

## AUTHOR CONTRIBUTIONS

**Yun Lin:** Conceptualization (equal); data curation (lead); funding acquisition (equal); investigation (lead); methodology (lead); project administration (lead); resources (equal); software (lead); supervision (equal); validation (equal); writing – original draft (lead); writing – review and editing (lead). **Dayu Liu:** Supervision (supporting). **Xuexin Li:** Resources (supporting). **Yan Ma:** Resources (supporting). **Xinliang Pan:** Conceptualization (lead); data curation (lead); funding acquisition (lead); investigation (lead); project administration (lead); resources (lead); supervision (lead); validation (lead); writing – original draft (lead); writing – review and editing (lead).

## CONFLICT OF INTEREST

The authors confirm that there are no conflicts of interest.

## Data Availability

The data that support the findings of this study are available from the corresponding author upon reasonable request.
